# Sequential Ion-Exchange Polishing of Oil Palm Trunk-Derived XOS-Containing Liquor: Effects of Holding Time and Water Washing on Sugar Recovery and Impurity Removal

**DOI:** 10.1021/acsomega.6c03824

**Published:** 2026-07-03

**Authors:** Che Engku Noramalina Che Engku Chik, Yitong Niu, Rozi Nuraika Ramli, Joo Shun Tan, Chee Keong Lee

**Affiliations:** † Bioprocess Technology Division, School of Industrial Technology, 26689 Universiti Sains Malaysia, Gelugor, Pulau Pinang 11800, Malaysia; ‡ Renewable Biomass Transformation Cluster, School of Industrial Technology, Universiti Sains Malaysia, Gelugor, Pulau Pinang 11800, Malaysia; § Malaysia Institute of Pharmaceuticals and Nutraceuticals, National Institutes of Biotechnology Malaysia, Halaman Bukit Gambir, Gelugor, Penang 11700, Malaysia

## Abstract

Downstream purification of lignocellulosic XOS-containing hydrolyzates requires effective impurity removal while minimizing the loss of the sugar-containing fraction. In this study, oil palm trunk (OPT)-derived XOS-containing liquor was purified by activated carbon pre̵-purification followed by sequential anion- and cation-exchange treatment, with emphasis on the effects of ion-exchange holding time and post-elution water washing on reducing sugar recovery and impurity removal. Activated carbon acted as the primary cleanup step, whereas ion exchange served as a polishing step for the partially purified fraction. In the ion-exchange study, holding time markedly affected recovery, with 10 min giving the highest reducing sugar recovery after both anion exchange (57.92%) and cation exchange (14.20%) while maintaining high impurity removal. Under this optimized holding time, post-elution washing with 1 L distilled water further increased recovery to 94.03% after anion exchange and 92.26% after cation exchange, while preserving substantial removal of furfural, acid-soluble lignin (ASL), and hydroxymethylfurfural (HMF). Application of the optimized downstream sequence at larger scale retained 72.18% of the reducing sugars and achieved final furfural, ASL, and HMF removals of 98.03, 98.73, and 98.76%, respectively. However, HPLC analysis showed that the final purified fraction remained xylose-dominant rather than DP2–DP6 XOS-dominant, indicating that downstream polishing improved cleanup and sugar recovery but could not overcome the saccharide profile generated during upstream hydrolysis. These findings establish a recovery-conscious polishing strategy for OPT-derived XOS-containing liquor and highlight the need to couple downstream purification with upstream hydrolysis control to improve final XOS selectivity.

## Graphical abstract

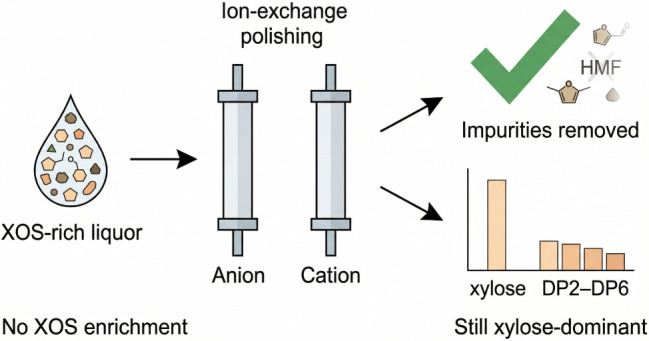

## 1 Introduction

Xylooligosaccharides (XOS) are value-added hemicellulose-derived oligosaccharides that have attracted considerable interest because of their functional and prebiotic properties.^1–3^ Among the different XOS fractions, low-degree-of-polymerization species, particularly DP2-DP6, are generally regarded as the most desirable components for food and nutraceutical applications.^4,5^ Lignocellulosic biomass provides a renewable xylan source for XOS production, and thermochemical and enzymatic processing routes have been widely investigated for converting agricultural residues into XOS-containing liquors.^6–8^ However, crude lignocellulosic hydrolyzates are compositionally complex and commonly contain monosaccharides, residual oligosaccharides, organic acids, furan derivatives, phenolic compounds, and soluble lignin-derived materials in addition to the target sugar fraction.^9–11^ Therefore, downstream purification is not merely a finishing step, but a critical operation that determines product usability, impurity burden, and sugar recovery.

Oil palm trunk (OPT) is an abundant lignocellulosic residue and a promising substrate for XOS production because of its hemicellulose-rich composition and ready availability from plantation renewal.^12–14^ Similar to other agricultural residues, OPT can be converted into XOS-containing hydrolyzates through thermochemical and enzymatic routes, but the resulting liquor is typically a complex mixture rather than a purified oligosaccharide stream.^15,16^ In addition to target oligosaccharides, such hydrolyzates may contain monosaccharides, furfural, hydroxymethylfurfural (HMF), soluble lignin-derived materials, organic acids, and other nontarget components that can reduce product quality and complicate downstream processing.^17–20^ Therefore, an effective downstream strategy is required not only to recover the sugar-containing fraction, but also to remove residual impurities without excessive sugar loss.^21,22^

Activated carbon treatment is widely used as a pre-purification step for lignocellulosic hydrolyzates because it can adsorb colored, aromatic, degradation-derived, and lignin-derived compounds while allowing subsequent recovery of sugar-containing fractions by aqueous or ethanol-assisted elution.^23–25^ Nevertheless, activated carbon alone may not provide sufficient polishing when residual acids, salts, charged degradation products, and soluble lignin-derived species remain in the liquor.^21^ Ion-exchange treatment is therefore a relevant refining step for carbohydrate-rich process streams, as anion- and cation-exchange resins can remove oppositely charged impurities, demineralize sugar liquors, and reduce residual organic acids or color-forming compounds.^26–29^ For XOS-containing liquors, the target oligosaccharides are largely non ionic, whereas many undesired compounds are more likely to interact with ion-exchange media. However, this separation is not governed by charge alone; resin contact time, feed pH, diffusion within the resin bed, elution flow behavior, and post-elution washing can all influence the trade-off between impurity removal and sugar retention.^30–32^ In particular, insufficient holding time may limit impurity adsorption, whereas excessive holding may increase sugar retention within the resin system. Despite this practical importance, the effects of ion-exchange holding time and post-elution water washing on OPT-derived XOS-containing liquor have not been systematically clarified.

Accordingly, this study investigated downstream purification of OPT-derived XOS-containing liquor with emphasis on recovery-conscious polishing. Activated carbon pre-purification was first evaluated to obtain a partially purified sugar-containing fraction. This fraction was then subjected to sequential anion- and cation-exchange treatment, with particular attention to the effects of holding time and post-elution water washing on reducing sugar recovery and removal of furfural, acid-soluble lignin, and HMF. The optimized downstream sequence was further assessed at larger scale to evaluate stagewise purification performance. In addition, HPLC-based saccharide profiling was used to determine whether downstream polishing altered the xylose/DP2–DP6 distribution of the final fraction. Through this approach, the study establishes a recovery-conscious downstream polishing strategy for OPT-derived XOS-containing liquor while clarifying that final DP2–DP6 XOS selectivity remains constrained by upstream hydrolysis.

## 2 Materials and Methods

### 2.1 Materials

Oil palm trunk (OPT) logs obtained from SKY Nutraceuticals Sdn. Bhd. were used as the lignocellulosic feedstock. The logs were cleaned to remove dirt and visible fungal contamination, cut into smaller pieces, and dried in a hot-air oven (BINDER BD115, Germany) at 80 °C for 3 d. The dried material was then ground (RIKEN) using a 3 mm sieve and further dried at 80 °C for 2 d. The resulting OPT fiber was characterized for pH using a pH meter (EUTECH PC700 Instruments, Australia) and for moisture content using a moisture analyzer (AND MX-50, Japan).^33^

### 2.2 Pretreatment and Enzymatic Hydrolysis

As shown in Scheme 1, OPT fiber was processed through sequential alkaline-assisted thermal treatment and enzymatic hydrolysis to generate an XOS-containing liquor, followed by activated carbon pre-purification and sequential ion-exchange polishing. OPT fiber was soaked in 0.5% NaOH solution at a solid-to-liquid ratio of 1:10 for 16–18 h. Excess alkali solution was removed, and the soaked fiber was then autoclaved (TOMY ES-315, Japan) at 121 °C for 60 min. Autohydrolysate was collected by manually squeezing the treated fiber. The pH of the autohydrolysate after autoclaving was 6.37 and was adjusted to 4.5 using 2 M HCl before enzymatic hydrolysis.

**Scheme 1:**
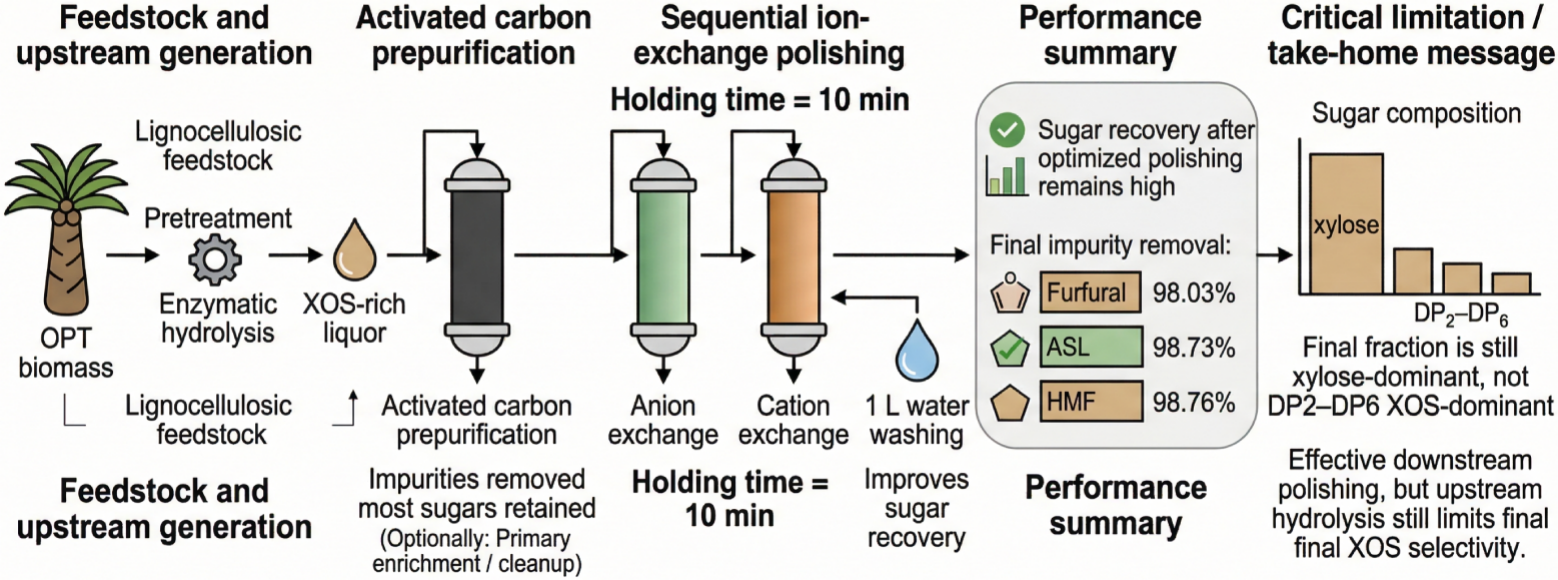
Integrated Purification Workflow for XOS-Containing Liquor Using Activated Carbon Pre-purification and Sequential Ion-Exchange Polishing

Enzymatic hydrolysis was conducted at 40 °C for 20 h using xylanase at an enzyme loading of 1 U per 600 mg hemicellulose. The resulting XOS-containing lysate was filtered before downstream purification.

### 2.3 Pilot-Scale Downstream Purification System

A pilot-scale downstream purification system was developed based on the laboratory-scale purification scheme. The system comprised two sample vessels (20 cm diameter × 30 cm height; 9.4 L each), one activated carbon vessel (36 cm diameter × 54 cm height; 55 L), one ethanol vessel (30 cm diameter × 36 cm height; 30.5 L), one distilled water vessel (30 cm diameter × 36 cm height; 30.5 L), one anion-exchange column (20 cm diameter × 30 cm height; 9.4 L), and one cation-exchange column (90 cm diameter × 12 cm height; 13.5 L), together with pumps, flowmeters, valves, and connecting hoses.

Granular activated carbon was pretreated with 0.2 M HCl at a 1:2 ratio, rinsed with deionized water until no further pH change was observed, and then loaded into the stainless-steel activated carbon vessel and left for 24 h before use.

### 2.4 Activated Carbon Purification

For optimization of activated carbon purification, each run used hydrolyzate obtained from 1 kg OPT fiber, corresponding to 2.9 L sample volume. The hydrolyzate was transferred to the activated carbon vessel and retained for 30 min before discharge of the unbound fraction. The adsorbed fraction was subsequently eluted using four successive 10 L cycles of ethanol–water eluent with a gradient up to 30% ethanol. Collected fractions were concentrated using a rotary evaporator at 74 °C to approximately 50% of their original volume before further analysis.

The activated carbon optimization examined the effects of ethanol gradient concentration, ethanol flow rate, and elution flow rate on purification performance. In the optimized larger-scale run, hydrolyzate derived from 5 kg OPT fiber, corresponding to 16 L, was retained in the activated carbon vessel for 30 min, the waste stream was discharged at 1.5 L/min, and the retained sugar fraction was eluted using seven successive 10 L cycles of 0–30% ethanol gradient at 5.1 L/min.

### 2.5 Ion-Exchange Purification

The partially purified fraction obtained after activated carbon treatment was filtered through an 11 μm membrane and subjected to sequential ion-exchange purification. The sample was passed first through an anion-exchange column packed with Amberlite IRA-67 (Alfa Aesar) and then through a cation-exchange column packed with Amberlite IR120H (J&K). The pH of the filtered feed entering the ion-exchange step was 4.41. No additional pH adjustment was applied before ion-exchange treatment so that the polishing step could be evaluated under the actual pH condition of the activated-carbon-prepurified liquor.

To evaluate the effect of contact time on purification performance, five holding durations were investigated: 0, 1, 5, 10, and 15 min. These holding times were selected to cover no-holding operation, short contact, intermediate contact, and extended contact within the practical operating window of the pilot-scale ion-exchange system. After sample elution, each ion-exchange column was rinsed with 1 L distilled water to recover residual reducing sugars retained in the resin bed. The collected fractions were concentrated by rotary evaporation before analysis.

After optimization, the downstream purification process was repeated under the selected condition using hydrolyzate produced from 5 kg OPT fiber (16 L).

### 2.6 Chemical Composition Analysis of OPT Fiber

The chemical composition of OPT fiber, including extractives, holocellulose, cellulose, hemicellulose, lignin, and ash, was determined using standard procedures. Extractives were measured according to TAPPI T204, with ethanol–toluene used in place of ethanol–benzene. Holocellulose and cellulose were determined according to TAPPI T249. Klason lignin was determined according to TAPPI T222, and ash was determined according to AOAC (2000). Hemicellulose content was calculated as the difference between holocellulose and cellulose contents. The hemicellulose content was calculated according:

1 Hemicellulose(%)=Holocellulose(%)−Cellulose(%)

The recovery of hemicellulose remaining in the autoclaved fiber residue was calculated according:

2 $$Recovery\;of\;hemicellulose\;in\;waste\;(\%)=\frac{Hemicellulose\;in\;fiber\;waste\;(g)}{Hemicellulose\;in\;OPTfiber\;(g)}\times 100\%$$

The apparent amount of hemicellulose released during pretreatment was calculated according:

3 Hemicellulosereleased(g)=HemicelluloseinOPTfiber(g)−Hemicelluloseinfiberwaste(g)

The apparent hemicellulose recovery in the autohydrolysate was calculated according:

4 $$Apparent\;hemicellulose\;recovery\;(\%)=\frac{Hemicellulose\;in\;OPTfiber\;(g)-Hemicellulose\;in\;fiber\;waste\;(g)}{Hemicellulose\;in\;OPTfiber\;(g)}\times 100$$

### 2.7 Reducing Sugar Determination

Reducing sugar concentration was determined by the 3,5-dinitrosalicylic acid (DNS) method according to Miller. Xylose (Merck, United States) was used to prepare the calibration curve. Briefly, 0.5 mL of sample was mixed with 2 mL DNS reagent (Sigma-Aldrich) and 1 mL distilled water in a 20 mL test tube. The mixture was boiled for 5 min, cooled to ambient temperature, and the absorbance was measured at 540 nm using a spectrophotometer (HACH DR2800, USA). A reagent blank was prepared in the same way, except that distilled water was used instead of sample.

### 2.8 Furfural Determination

Furfural concentration was determined spectrophotometrically using a furfuraldehyde calibration curve. For sample analysis, 2.5 mL of sample was diluted with 25 mL distilled water, and acetonitrile was added to a final volume of 50 mL in a volumetric flask. Absorbance was measured at 280 nm using a UV–vis spectrophotometer (Shimadzu UV-1800, Japan).

### 2.9 Acid-Soluble Lignin Determination

Acid-soluble lignin (ASL) was determined according to the method of Maekawa et al. Absorbance was measured at 205 nm using a UV–vis spectrophotometer (Shimadzu UV-1800, Japan) with a 1 cm light-path cuvette. A 4% H_2_SO_4_ solution was used as the blank. Samples were diluted to give absorbance values between 0.2 and 0.7, and the blank was diluted at the same ratio as the sample. ASL concentration was calculated according:

5 $$ASL\left(\frac{g}{L}\right)=\frac{A\times df}{a\times b}$$

where *A* is the absorbance at 205 nm, *df* is the dilution factor, *A* is the absorptivity coefficient taken as 110 L/g·cm, and *b* is the cell path length (1 cm).

### 2.10 Hydroxymethylfurfural Determination

Hydroxymethylfurfural (HMF) was determined according to Adu et al., with slight modification. A stock solution of 2 mg/mL HMF was prepared by dissolving 10 mg in 5 mL deionized water. Working standards were prepared by diluting the stock solution with deionized water to lower concentrations. Absorbance was measured at 284 nm using a UV–vis spectrophotometer (Shimadzu UV-1800, Japan), with deionized water used as the blank. Sample concentrations were obtained from the calibration curve.

### 2.11 Ethanol Removal

Residual ethanol in collected fractions was removed either by rotary evaporation using a Laborota 400 rotary evaporator (Heidolph) or by oven drying, depending on the purification stage.

### 2.12 HPLC Analysis of XOS

XOS composition was analyzed by HPLC. Samples were diluted 20-fold and filtered through a 0.22 μm membrane prior to injection. Xylose standard (Merck, United States) was prepared at 6 mg/mL. Commercial XOS standard (Shandong Longlive Biotechnology, China) was prepared at the same concentration. Additional XOS standards, including xylobiose (DP2), xylotriose (DP3), xylotetraose (DP4), xylopentaose (DP5), and xylohexaose (DP6), were obtained from Megazyme (Ireland).

The HPLC system consisted of a Shimadzu LC-20AD equipped with an RID-10A refractive index detector, CTO-10AS VP column oven, SIL-20A HT autosampler, and DGU-20A5 Prominence degasser. Data acquisition was performed using LC Solution software. Separation was carried out on a Rezex RSO oligosaccharide column (200 × 10.00 mm) at 75 °C, with the detector maintained at 60 °C. Deionized water was used as the mobile phase at a flow rate of 0.3 mL/min.

Quantification of xylose and DP2–DP6 XOS was performed using an external standard approach. Individual xylose and DP2–DP6 standards were analyzed under the same chromatographic conditions as the process samples, and peak assignment was performed by matching the retention times of sample peaks with those of the authentic standards. The chromatograms of the xylose and DP2–DP6 standards are provided in Figure S1, and their retention times are summarized in Table S1. The commercial XOS reference was also analyzed to confirm the practical elution order of the oligomeric fractions (Figure S2). Representative chromatograms of OPT-derived process streams are provided in Figure S3. Because authentic standards were used for the quantified saccharides, compound-specific external standard quantification was applied, and no universal response correction factor was used. Full HPLC peak-assignment and quantification results are provided in Table S2. The HPLC quantification focused on xylose and DP2–DP6 XOS detectable against the available external standards; higher-DP soluble oligomers and nonmonomeric carbohydrate fractions were not quantified by liquid-fraction acid hydrolysis in the present study. The XOS purity of the purified fraction was calculated according:

6 $$XOS\;purity\;(\%)=\frac{C}{D}\times 100\%$$

where *C* is the mass of XOS in the purified sample and *D* is the total mass of the purified sample.

### 2.13 Recovery and Removal Calculations

Reducing sugar recovery at each purification stage was calculated according:

7 $$Reducing\;sugar\;recovery\;(\%)=\frac{Mass\;of\;reducing\;sugar\;in\;collected\;purified\;fraction}{Mass\;of\;reducing\;sugar\;in\;feed\;stream}\times 100\%$$

Impurity removal for furfural, ASL, and HMF was calculated according:

8 $$Impurity\;removal\;(\%)=\frac{Impurity\;mass\;removed\;during\;purification}{Initial\;\mathrm{imp}urity\;mass}\times 100\%$$

Where impurity mass removed during purification was calculated from the difference between the initial impurity mass in the feed stream and the residual impurity mass in the purified fraction. The impurity refers to furfural, ASL, or HMF. If the yield of XOS over the initial OPT feedstock is reported, it was calculated according:

9 $$XOS\;yield\;over\;OPT\;feedstock\;(\%)=\frac{mass\;of\;XOS\;recovered}{mass\;of\;initial\;OPT\;fiber}\times 100\%$$

### 2.14 Statistical Analysis

All measurements were performed in triplicate (n = 3) unless otherwise stated, and results are expressed as mean ± standard deviation (SD). Statistical analysis was performed using one-way analysis of variance (ANOVA), followed by Tukey’s post hoc test. Differences were considered statistically significant at *p* < 0.05.

## 3 Results and Discussion

### 3.1 Performance of Activated Carbon Pre-purification

The transfer of the activated carbon purification step from laboratory scale to the fabricated pilot-scale system initially resulted in poor sugar recovery under the control condition. Only 1.94% of the reducing sugars were recovered after activated carbon elution, although furfural, ASL, and HMF removals remained high at 97.53, 97.76, and 96.39%, respectively. This result indicates that the initial pilot-scale condition retained the target sugar fraction effectively on the activated carbon bed but did not provide sufficiently efficient desorption for recovery of the adsorbed sugars.^9,34^

To improve the pre-purification step, the activated carbon process was optimized by varying the ethanol gradient concentration, ethanol flow rate, and elution flow rate while maintaining a fixed binding time of 30 min. As summarized in Table 1 and shown in Figure 1a, sugar recovery was highly dependent on the elution conditions. Among the optimized conditions, Condition 1 gave the highest sugar recovery, reaching 68.14%, whereas Conditions 2–4 resulted in substantially lower recoveries of 35.38, 32.86, and 32.73%, respectively. Recovery increased again under Condition 5 to 51.38%, although it remained lower than that obtained under Condition 1. These results show that efficient desorption of the activated-carbon-bound fraction required not only an appropriate ethanol gradient but also suitable hydrodynamic conditions during elution.^35^

**Table 1:** Activated Carbon Pre-purification Conditions and Corresponding Purification Performance^a^

No	Ethanol gradient concentration (%)	Elution mode	Ethanol flow rate (L/min)	Elution flow rate (L/min)	Binding time (min)	Sugar recovery (%)	Furfural removal (%)	ASL removal (%)	HMF removal (%)
Control	0–30	Continuous, total 30 L	—	0.52	30	1.94	97.53	97.76	96.39
1	30	10 L × 4	5.1	5.1	30	68.14	99.14	99.70	99.19
2	40	10 L × 4	5.0	1.5	30	35.38	99.54	99.82	99.55
3	30	10 L × 4	4.7	4.7	30	32.86	98.75	98.24	97.67
4	30	10 L × 4	4.7	4.0	30	32.73	98.91	97.24	98.75
5	30	10 L × 4	4.7	5.1	30	51.38	94.58	93.34	96.38

a Notes: Sugar recovery refers to cumulative reducing sugar recovery after activated carbon elution. Impurity removal values refer to total removal of furfural, ASL, and HMF in the activated-carbon-purified fraction. The control condition corresponds to the initial pilot-scale transfer of the laboratory-scale purification scheme, whereas Conditions 1–5 correspond to subsequent optimization experiments.

**Figure 1:**
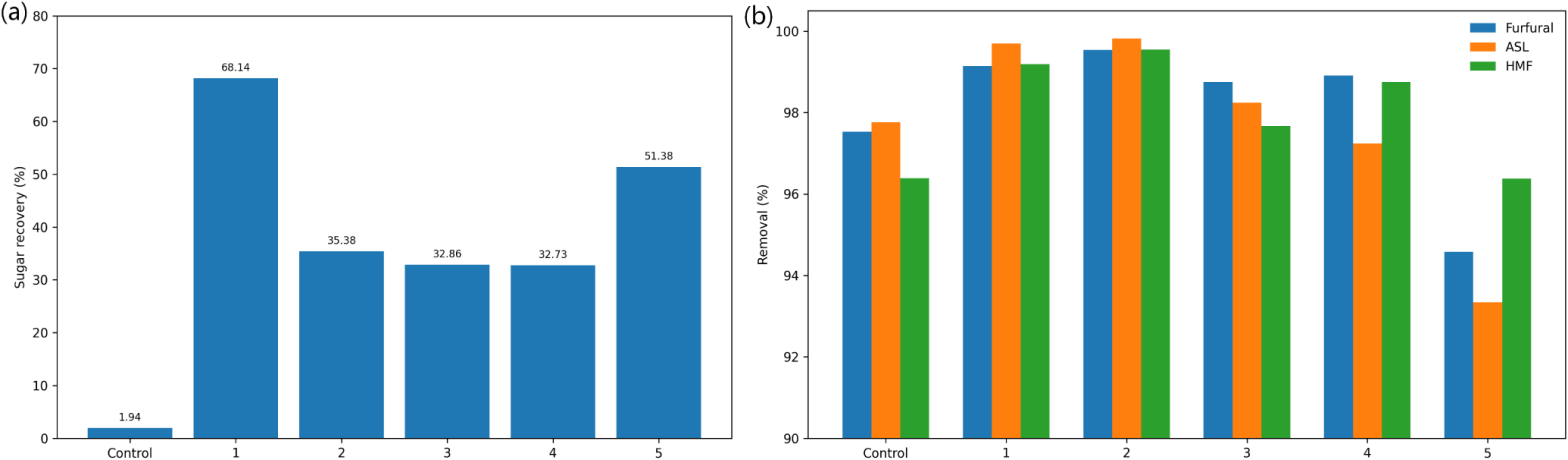
Recovery and impurity removal during activated carbon pre-purification: (a) sugar recovery and (b) impurity removal.

A different trend was observed for impurity removal. As shown in Figure 1b, all optimized conditions achieved high removals of furfural, ASL, and HMF, confirming that activated carbon functioned effectively as a primary cleanup step for degradation-derived compounds. The highest overall impurity removal was observed under Condition 2, with furfural, ASL, and HMF removals of 99.54, 99.82, and 99.55%, respectively. By comparison, Condition 1 provided slightly lower impurity removal but substantially higher sugar recovery. This contrast demonstrates a clear recovery–removal trade-off during activated carbon pre-purification: conditions that favored stronger impurity removal did not necessarily maximize sugar recovery, whereas conditions that improved sugar recovery did not always produce the highest purification efficiency.^36,37^

Taken together, these results confirm that activated carbon was effective as a pre-purification step for concentrating the sugar-containing fraction and substantially reducing major impurities, but its performance depended strongly on the elution conditions. Under the optimized conditions, activated carbon served as an effective primary enrichment and cleanup step for the OPT-derived liquor. Nevertheless, the pre-purified fraction still required further polishing to remove residual impurities and improve downstream product quality.^38^ The activated-carbon-treated liquor was therefore subjected to sequential anion- and cation-exchange purification in the subsequent step.

### 3.2 Effect of Ion-Exchange Holding Time on Sugar Recovery and Impurity Removal

The holding time applied during ion-exchange purification markedly influenced the recovery of the sugar-containing fraction. Prior to ion exchange, the activated-carbon-purified liquor was filtered and had a pH of 4.41. Because XOS is a non-ionic sugar, the ion-exchange resins were intended primarily to remove charged impurities rather than the target oligosaccharide fraction itself.^26,27^ The feed pH is nevertheless an important factor for ion-exchange polishing because it can affect the ionization state of acidic degradation products, soluble lignin-derived compounds, and functional groups on the resin surface. Therefore, the observed recovery–removal balance should be interpreted as the performance of the resin sequence at pH 4.41 rather than as a pH-optimized condition. As shown in Figure 2a and Table 2, the sugar recovery after the anion-exchange step varied substantially with holding time. Recovery remained relatively low at 0–5 min, increased sharply to 57.92% at 10 min, and then decreased drastically to 3.82% at 15 min. After the cation-exchange step, the same general trend was observed, with the highest recovery also recorded at 10 min (14.20%). These results indicate that 10 min provided the most favorable operating window for ion-exchange polishing under the present conditions.

**Figure 2:**
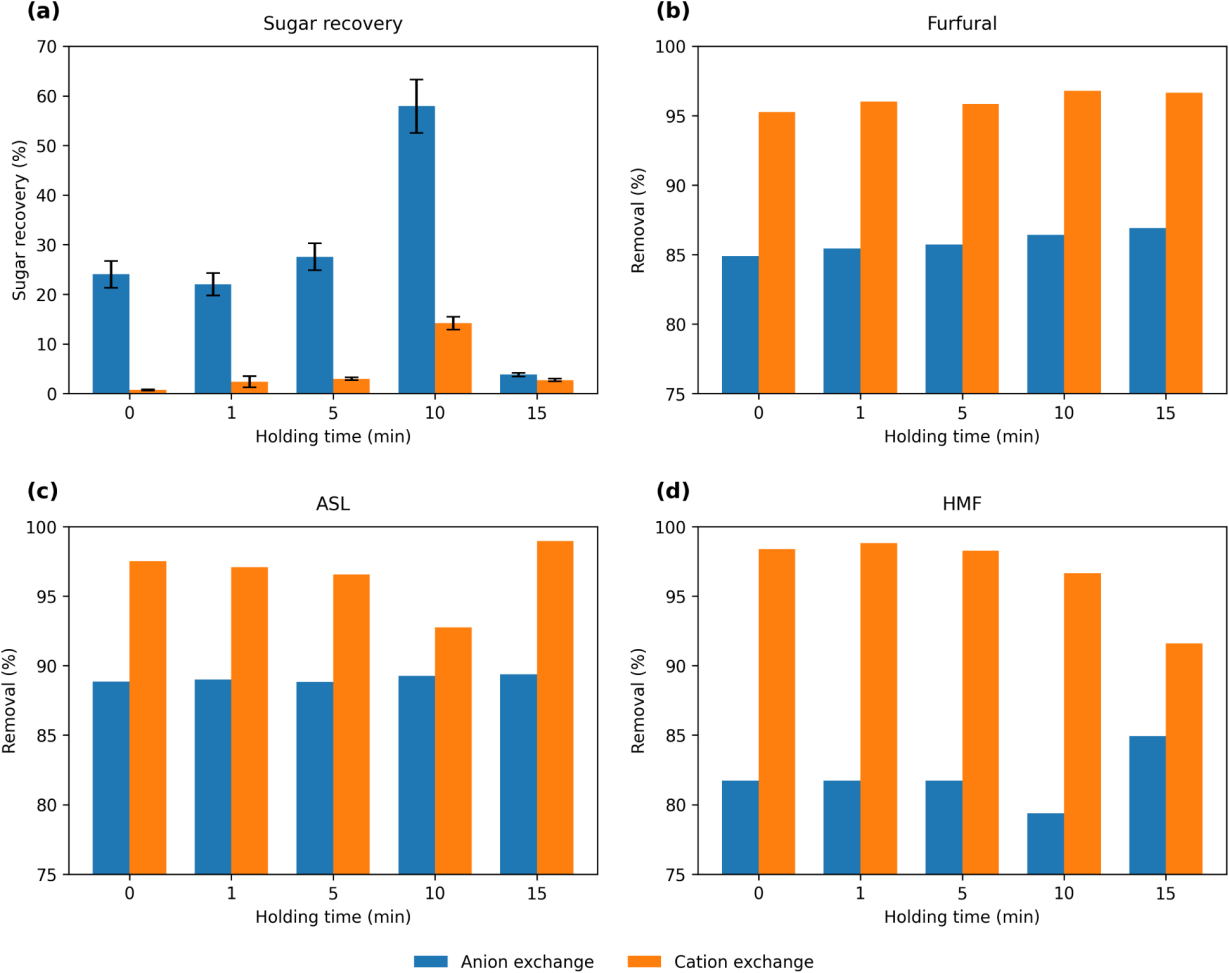
Effect of holding time during ion-exchange purification: (a) sugar recovery, (b) furfural removal, (c) ASL removal, and (d) HMF removal.

**Table 2:** Effect of Holding Time on Sequential Ion-Exchange Purification of Partially Purified XOS Liquor^a^

Holding time (min)	Fraction	RS (g)	Recovery (%)	Furfural removed (g)	Removal (%)	ASL removed (g)	Removal (%)	HMF removed (g)	Removal (%)
—	Filtered partially purified feed	10.16 ± 0.94	100.00	0.18 ± 0.02	0.00	0.11 ± 0.01	0.00	0.03 ± 0.00	0.00
0	Anion exchange	2.44 ± 0.16	24.02	0.15 ± 0.00	84.89	0.09 ± 0.00	88.85	0.02 ± 0.00	81.72
0	Cation exchange	0.08 ± 0.01	0.74	0.17 ± 0.00	95.27	0.10 ± 0.00	97.51	0.02 ± 0.00	98.40
1	Anion exchange	2.24 ± 0.10	22.05	0.15 ± 0.00	85.45	0.09 ± 0.00	89.00	0.02 ± 0.00	81.72
1	Cation exchange	0.24 ± 0.11	2.38	0.17 ± 0.00	96.01	0.10 ± 0.00	97.08	0.03 ± 0.03	98.81
5	Anion exchange	2.80 ± 0.10	27.56	0.15 ± 0.00	85.72	0.10 ± 0.00	88.83	0.02 ± 0.03	81.72
5	Cation exchange	0.30 ± 0.00	2.97	0.17 ± 0.00	95.84	0.10 ± 0.00	96.56	0.03 ± 0.03	98.26
10	Anion exchange	5.88 ± 0.01	57.92	0.15 ± 0.00	86.41	0.09 ± 0.00	89.25	0.02 ± 0.03	79.38
10	Cation exchange	1.44 ± 0.01	14.20	0.17 ± 0.00	96.79	0.10 ± 0.00	92.76	0.03 ± 0.00	96.65
15	Anion exchange	0.39 ± 0.01	3.82	0.16 ± 0.00	86.90	0.09 ± 0.00	89.37	0.02 ± 0.00	84.93
15	Cation exchange	0.28 ± 0.01	2.72	0.17 ± 0.00	96.64	0.10 ± 0.00	98.97	0.03 ± 0.00	91.61

a Notes: The filtered partially purified feed was used as the common baseline for all holding-time experiments. RS, reducing sugars; ASL, acid-soluble lignin; HMF, hydroxymethylfurfural. The ion-exchange holding times investigated were 0, 1, 5, 10, and 15 min. Values are reported as mean ± SD.

The effect of holding time can be interpreted in terms of contact between the sample and the resin bed. When a concentrated partially purified liquor is introduced into a column containing the stationary resin, the charged impurities require sufficient time to interact with the available exchange sites. At very short holding times, the contact period may be insufficient for effective adsorption of ionic impurities to the resin. In contrast, prolonged holding may increase the retention of loosely associated sugar components within the resin bed. As discussed in the original analysis, adsorption behavior depends on the available surface area, the chemical nature of the resin and solutes, the charges carried by the liquid-phase impurities and the solid resin, and the rate of diffusion within the system.^39–41^ From this perspective, the sharp drop in recovery at 15 min suggests that excessive residence time promoted the retention of weakly bound sugar species, thereby reducing the recovered fraction.

Compared with the strong variation observed for sugar recovery, impurity removal remained consistently high across the investigated holding times. As shown in Figure 2b–d and Table 2, furfural removal after the anion-exchange step ranged from 84.89 to 86.90%, while the corresponding cation-exchange values ranged from 95.27 to 96.79%. ASL removal after anion exchange remained between 88.83 and 89.37%, and increased further after cation exchange to 92.76–98.97%. For HMF, the anion-exchange removal ranged from 79.38 to 84.93%, whereas the cation-exchange removal ranged from 91.61 to 98.81%. These results show that the cation-exchange column consistently provided an additional polishing effect after anion exchange, and that impurity removal was less sensitive to holding time than sugar recovery.^42^

Taken together, the holding-time experiment demonstrated that the critical trade-off in this ion-exchange step was not the ability to remove impurities, as high removal efficiencies were consistently achieved under all tested conditions, but rather the ability to maximize impurity removal while minimizing sugar loss. Based on this finding, a holding time of 10 min condition was selected as the optimal holding time because it provided the highest sugar recovery following both the anion- and cation-exchange steps while maintaining high furfural, ASL, and HMF removals. Moreover, the pronounced loss in recovery at 15 min supports the subsequent evaluation of post-elution water washing, as it suggests that part of the sugar fraction may have remained weakly retained in the resin bed and could potentially be recovered by additional elution. Although the selected holding-time range was sufficient to identify 10 min as the best condition within the tested pilot-scale operating window, additional intermediate and longer holding times would be useful in future work to further refine the residence-time profile and validate the robustness of sugar recovery behavior.

### 3.3 Effect of Post-elution Water Washing under the Optimized Holding Time

After identifying 10 min as the optimal holding time, an additional post-elution water-washing step was evaluated to improve recovery from the ion-exchange columns. In this experiment, 1 L of distilled water was introduced after sample elution in two successive 500 mL portions and allowed to flow through the column into the collector. As shown in Table 3 and Figure S4, this additional elution step markedly increased the recovery of the sugar-containing fraction, reaching 94.03% after the anion-exchange step and 92.26% after the cation-exchange step. These values were substantially higher than those obtained in the holding-time experiment without additional washing, indicating that a large portion of the apparent sugar loss during ion exchange could be recovered by further elution.

**Table 3:** Effect of Post-elution Water Washing under the Optimized Ion-Exchange Condition^a^

Fraction	RS (g)	Recovery (%)	Furfural (g)	Removal (%)	ASL (g)	Removal (%)	HMF (g)	Removal (%)
Filtered partially purified feed	29.96 ± 0.94	100.00	0.54 ± 0.02	0.00	0.32 ± 0.01	0.00	0.09 ± 0.00	0.00
Anion exchange with post-elution water washing	28.17 ± 0.74	94.03	0.03 ± 0.01	93.88	0.18 ± 0.00	42.90	0.02 ± 0.00	72.48
Cation exchange with post-elution water washing	27.64 ± 0.16	92.26	0.02 ± 0.00	96.59	0.07 ± 0.00	76.90	0.02 ± 0.00	80.68

a Notes: The optimized ion-exchange condition corresponded to a holding time of 10 min. Post-elution water washing was carried out with 1 L distilled water, applied as two successive 500 mL portions. RS, reducing sugars; ASL, acid-soluble lignin; HMF, hydroxymethylfurfural. Values are reported as mean ± SD.

The improvement in recovery can be interpreted in relation to the nature of the XOS fraction present in the partially purified liquor. As discussed in the original analysis, the sample may contain both lignin-associated XOS (XOS-lignin) and “free” XOS. The low recovery observed before additional washing was probably due, at least in part, to the retention of XOS-lignin on the resin. For the free XOS fraction, the separation behavior may also depend on the degree of polymerization (DP), with higher-DP oligosaccharides being more susceptible to retention within the resin system. From this perspective, the post-elution water wash did not merely dilute the column contents, but displaced sugar components that had remained weakly retained after the main elution step.

Despite the pronounced increase in recovery, impurity removal remained substantial after water washing. Furfural removal reached 93.88% after anion exchange and increased further to 96.59% after cation exchange. A similar improvement was observed for ASL and HMF, with ASL removal increasing from 42.90 to 76.90% and HMF removal increasing from 72.48 to 80.68% after the cation-exchange step. These results indicate that the additional water wash did not eliminate the cleanup effect of the ion-exchange resins. Instead, the cation-exchange column continued to provide further polishing of the partially purified liquor after anion exchange.

The present findings are consistent with previous observations that extensive rinsing with distilled water after ion-exchange treatment can improve XOS recovery while maintaining effective impurity removal. Corbett et al. reported that water washing after resin treatment improved the recovery of XOS for prebiotic applications, and the same tendency was observed here. Therefore, the combination of a 10 min holding time and 1 L postelution water washing was selected as the optimized ion-exchange polishing condition. This result further suggests that part of the sugar loss observed in the earlier holding-time study arose from reversible retention within the resin bed rather than irreversible loss of the target fraction.

### 3.4 Performance and Limitations of the Optimized Downstream Polishing Sequence

After optimization of the downstream purification conditions, the process was applied at the larger scale using hydrolyzate derived from 5 kg OPT fiber. As summarized in Table 4 and Figure 3, the optimized sequence, consisting of activated carbon pre-purification followed by sequential anion- and cation-exchange treatment, provided progressive cleanup of the sugar-containing fraction while maintaining an overall reducing sugar recovery of 72.18% in the final cation-exchange fraction. Activated carbon served as the primary enrichment step, increasing impurity removal to 94.76% for furfural, 93.57% for ASL, and 97.19% for HMF in the eluted fraction, while retaining 85.19% of the reducing sugars. Subsequent ion-exchange polishing further improved impurity removal, and the final cation-exchange fraction reached 98.03, 98.73, and 98.76% removal for furfural, ASL, and HMF, respectively. These results confirm that the optimized downstream sequence was effective for stepwise purification of the OPT-derived liquor.

**Table 4:** Stagewise Purification Performance of the Optimized Downstream Polishing Sequence^a^

Process stream	Reducing sugars (g)	Recovery (%)	Furfural (g)	Removal (%)	ASL (g)	Removal (%)	HMF (g)	Removal (%)
XOS-rich enzymatic hydrolyzate	308.59 ± 1.09	100.00	7.63 ± 0.17	0.00	29.96 ± 0.77	0.00	8.89 ± 0.02	0.00
Activated-carbon-retained fraction	291.93 ± 0.32	94.52	1.82 ± 0.02	76.15	3.76 ± 0.11	87.45	1.15 ± 0.01	87.06
Activated-carbon-eluted fraction	263.13 ± 0.39	85.19	0.40 ± 0.01	94.76	1.93 ± 0.00	93.57	0.25 ± 0.01	97.19
Anion-exchange fraction	232.71 ± 1.43	75.35	0.26 ± 0.00	96.59	0.60 ± 0.00	98.00	0.17 ± 0.00	98.09
Cation-exchange fraction	222.93 ± 3.54	72.18	0.15 ± 0.00	98.03	0.38 ± 0.00	98.73	0.11 ± 0.00	98.76

a Notes: The optimized downstream polishing sequence comprised activated carbon pre-purification followed by sequential anion- and cation-exchange purification. Recovery values are reported relative to the enzymatic hydrolyzate feed. Values are reported as mean ± SD (n = 3).

**Figure 3:**
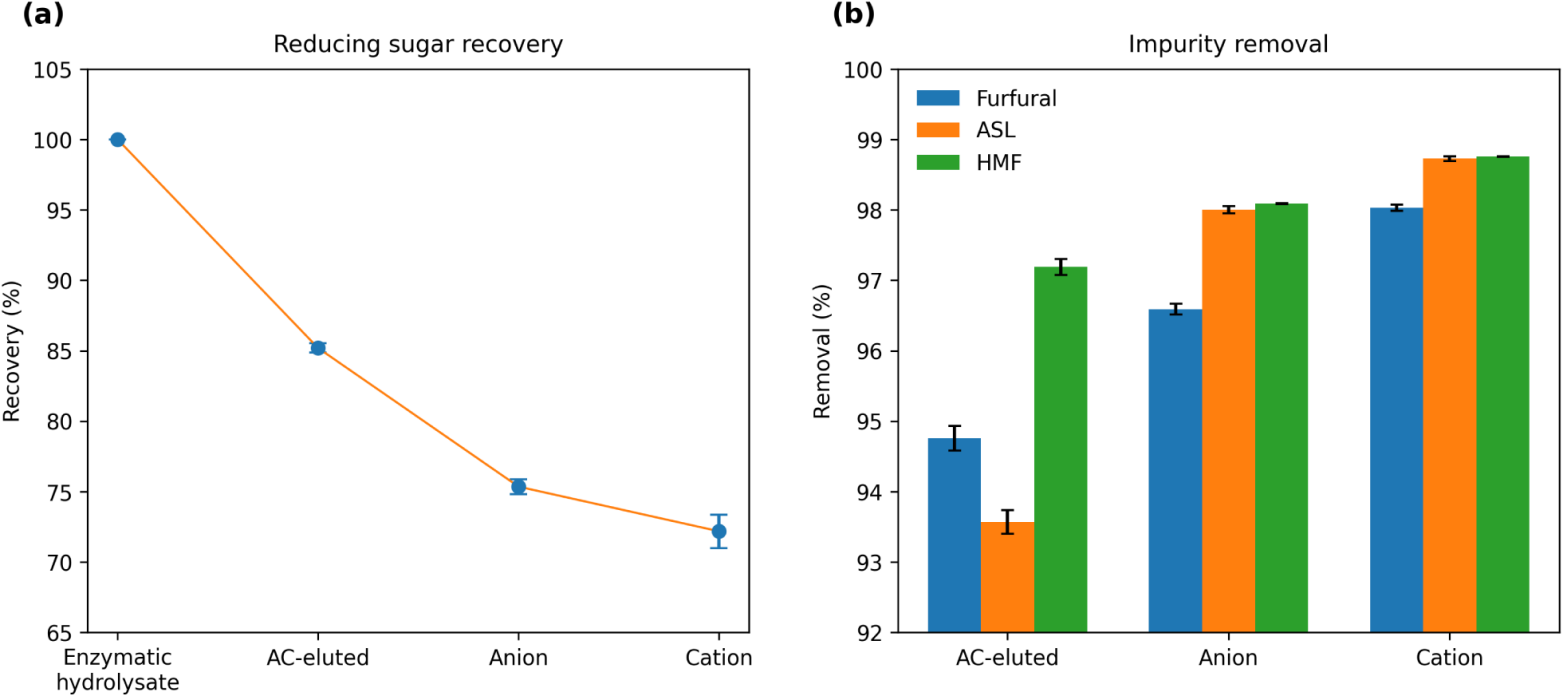
Stagewise performance of the optimized downstream polishing sequence: (a) reducing sugar recovery and (b) impurity removal.

The stagewise profiles also clarify the functional roles of the individual purification steps. Activated carbon removed a large proportion of degradation-derived and lignin-derived impurities while preserving most of the sugar fraction, indicating that this step was primarily responsible for enrichment and bulk cleanup. In contrast, the ion-exchange columns acted as polishing units. The anion-exchange step further reduced the residual impurity load after activated carbon treatment, and the cation-exchange step provided the highest overall impurity removal in the final fraction. This progressive increase in cleanup, together with the moderate decrease in reducing sugar recovery across the sequence, supports the interpretation that activated carbon and ion exchange performed complementary functions rather than redundant ones.

Despite the strong impurity-removal performance, HPLC analysis revealed an important limitation in the saccharide distribution of the final purified fraction. Peak assignment was based on retention-time matching with xylose and DP2–DP6 external standards analyzed under the same chromatographic conditions. The standard chromatograms and retention times are provided in Figure S1 and Table S1, while the commercial XOS reference and representative OPT-derived process-stream chromatograms are shown in Figures S2 and S3. The corresponding peak-assignment and quantification results are summarized in Table S2. As shown in Figure 4a, xylose remained the dominant quantified saccharide throughout the downstream sequence. In the final cation-exchange fraction, xylose was quantified at 195.745 g, whereas the total amount of DP2–DP6 XOS was 36.98 g. Figure 4b further shows that DP2 accounted for most of the quantified DP2–DP6 fraction, while DP3–DP6 remained comparatively low. Accordingly, although the final fraction contained only low residual nonsugar impurities, it remained xylose-dominant rather than DP2–DP6 XOS-dominant.

**Figure 4:**
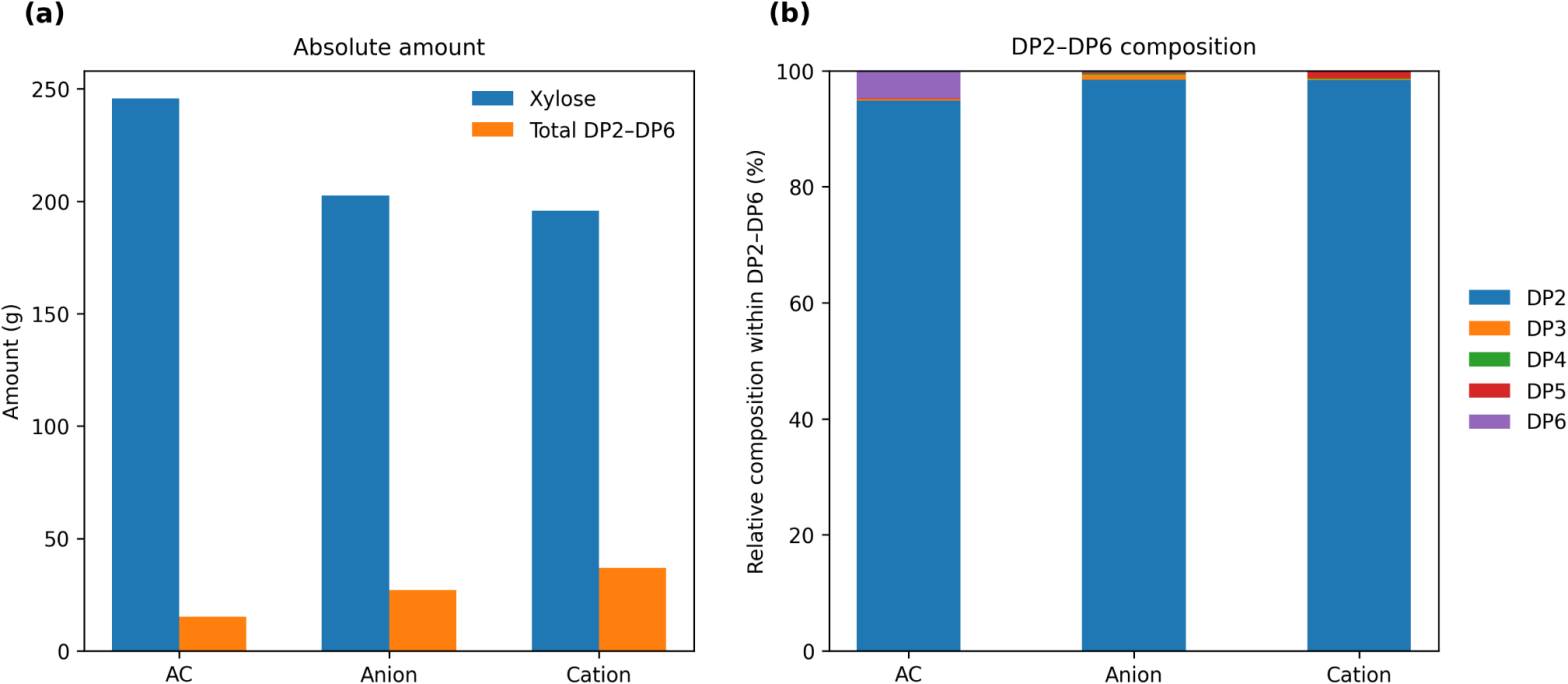
Saccharide distribution of OPT-derived purified fractions: (a) xylose and total DP2–DP6 XOS, and (b) relative DP2–DP6 composition.

The xylose-dominant profile can be mechanistically linked to the upstream hydrolysis conditions rather than to the polishing step itself. During alkaline-assisted thermal treatment, xylan is solubilized from OPT and partly converted into soluble xylo-oligosaccharides; during the subsequent enzymatic hydrolysis step, xylanase further cleaves xylan and longer-chain xylo-oligosaccharides. If hydrolysis proceeds beyond the window that favors DP2–DP6 accumulation, the oligosaccharide fraction can be further depolymerized to xylose. In the present process, enzymatic hydrolysis was conducted at 40 °C for 20 h after pH adjustment to 4.5. The original process records also indicated that the hydrolysis degree exceeded 50%, suggesting extensive cleavage of xylan-derived substrates. This is consistent with the HPLC results, where xylose increased markedly after activated carbon purification and remained the dominant quantified saccharide after sequential ion exchange. Therefore, the high xylose content in the final fraction is best interpreted as a consequence of upstream saccharide generation and depolymerization rather than selective formation during downstream purification.

These observations indicate that the final saccharide distribution was governed primarily by the upstream hydrolysis stage, whereas downstream polishing mainly altered impurity levels and sugar recovery. From this perspective, the optimized downstream sequence should be regarded as an effective cleanup and polishing strategy for OPT-derived hydrolyzate rather than a standalone solution for maximizing final XOS selectivity. Further improvement in product composition will require upstream control of hydrolysis severity, enzyme loading, hydrolysis duration, and pH buffering to favor DP2–DP6 accumulation while limiting excessive conversion to xylose. To place the present process in the context of reported XOS purification strategies, selected studies involving activated carbon, ion exchange, calcium-based treatment, membrane filtration, or their combinations are summarized in Table 5.

**Table 5:** Comparison with Reported Purification Strategies for XOS-Containing Liquors^a^

Feedstock	Purification strategy	Key reported performance	Main feature	Ref
Miscanthus hydrolyzate	AC adsorption/elution + serial ion exchange	45.4% XOS recovery from AC; 91.3% XOS recovery from serial ion exchange	AC + ion-exchange route	^38^
Wheat straw spent liquor	Fixed-bed ion-exchange resin	93.09% XOS recovery; 98.03% lignosulfonate recovery	Ion-exchange separation	^43^
Prehydrolysis liquor	Ca(OH)_2_ + AC treatment	66.9% lignin removal; 70.1% furfural removal; 5.9% xylosugar loss	Lignin/furfural cleanup	^44^
Bamboo XOS liquor	AC + ion exchange + membrane filtration	7.5% yield; 92.3% XOS purity; 98.9% total sugar proportion	Food-grade XOS refining	^22^
OPT-derived XOS-containing liquor	AC pre-purification + sequential ion exchange + water washing	72.18% reducing sugar recovery; 98.03% furfural, 98.73% ASL, and 98.76% HMF removal	Larger-scale OPT polishing; xylose-dominant final fraction	This work

a Notes: AC, activated carbon; ASL, acid-soluble lignin; HMF, hydroxymethylfurfural; OPT, oil palm trunk; XOS, xylooligosaccharides. Reported values are compared as stated in the original studies because recovery, purity, and impurity-removal bases differ among processes.

As shown in Table 5, reported purification strategies for lignocellulosic XOS-containing liquors have commonly relied on activated carbon, ion-exchange resin, calcium-based treatment, membrane filtration, or combined processes to improve XOS recovery, purity, or impurity removal. Compared with these studies, the present work is distinguished by the larger-scale treatment of OPT-derived liquor and by the explicit optimization of ion-exchange holding time and postelution water washing. The optimized process achieved high furfural, ASL, and HMF removals while retaining 72.18% of reducing sugars. However, unlike processes that produced XOS-dominant final products, the present fraction remained xylose-dominant, further confirming that downstream polishing must be coupled with upstream hydrolysis control to improve final DP2–DP6 XOS selectivity.

The process also requires consideration of solvent, water, and resin use. The activated carbon step used ethanol–water eluent, and the larger-scale run required seven 10 L elution cycles; therefore, ethanol recovery and reuse would be essential for improving greenness and reducing operating cost. The 1 L postelution water wash improved sugar recovery but increased aqueous effluent generation, indicating that water washing should be balanced with effluent handling and potential water recycling. Resin cost is also expected to depend on regeneration efficiency, reuse cycles, and adsorption-capacity stability. These factors should be included in future techno-economic and environmental evaluations of the process.

The downstream polishing performance should also be interpreted within the specific operating window used in this study. The ion-exchange step was conducted at the actual pH of the activated-carbon-prepurified liquor (pH 4.41), without independent pH adjustment. Because feed pH can influence the ionization state of acidic degradation products, soluble lignin-derived compounds, and resin functional groups, the recovery–removal balance observed here should be regarded as process performance at pH 4.41 rather than as a pH-optimized condition. In addition, resin regeneration, reuse, lifetime, and adsorption-capacity stability were not evaluated in the present work.^45,46^ These factors are important for assessing long-term operation and process economics and should be examined in future studies before the sequence is advanced toward industrial implementation. Future work may also consider application-specific modulation of residual phenolic content, because low phenolic levels could be beneficial in livestock-oriented phytogenic products.

Taken together, the optimized downstream polishing sequence demonstrated effective larger-scale operation and excellent impurity-removal capability, establishing activated carbon pre-purification followed by sequential ion exchange as a workable purification route for OPT-derived XOS-containing liquor. However, the final composition results also show that downstream optimization alone was insufficient to achieve a DP2–DP6 XOS-dominant final fraction under the present process configuration. This outcome defines both the value and the boundary of the current study: the work provides an effective recovery-conscious polishing strategy, while also identifying upstream saccharide generation as the remaining bottleneck for improving final XOS selectivity.

## 4 Conclusion

Activated carbon pre-purification followed by sequential anion- and cation-exchange treatment provided an effective downstream polishing strategy for OPT--derived XOS-containing liquor. Activated carbon acted as the primary enrichment and cleanup step, whereas ion exchange provided additional polishing of the partially purified fraction. Among the tested ion-exchange conditions, a 10 min holding time gave the best balance between reducing sugar recovery and impurity removal. Under this condition, post-elution water washing further improved reducing sugar recovery to 94.03% after anion exchange and 92.26% after cation exchange while maintaining substantial removal of furfural, ASL, and HMF. At larger scale, the optimized sequence retained 72.18% of the reducing sugars and achieved final furfural, ASL, and HMF removals of 98.03, 98.73, and 98.76%, respectively. These results show that recovery-conscious polishing of OPT-derived XOS-containing liquor can be achieved by combining activated carbon pre-purification with optimized ion-exchange operation. However, the final purified fraction remained xylose-dominant rather than DP2–DP6 XOS-dominant, indicating that downstream polishing alone is insufficient to maximize final XOS selectivity. Further improvement will therefore require coupling downstream purification optimization with upstream hydrolysis control to favor DP2–DP6 XOS accumulation and limit excessive xylose formation.

## Supplementary Material



## Notes

HPLC chromatograms of xylose and DP2–DP6 standards, commercial XOS reference, and OPT-derived process streams; evaluation of postelution water washing on sugar recovery and impurity removal; retention-time data and HPLC quantification of xylose and XOS in standards, commercial XOS, and process streams (PDF)

## Abbreviations

OPT: oil palm trunk
XOS: xylooligosaccharides
ASL: acid-soluble lignin
HMF: hydroxymethylfurfural
DNS: 3,5-dinitrosalicylic acid
HPLC: high-performance liquid chromatography
DP: degree of polymerization.
