# Supplementary material for: Sequential Ion-Exchange Polishing of Oil Palm Trunk-Derived XOS-Containing Liquor: Effects of Holding Time and Water Washing on Sugar Recovery and Impurity Removal
Source: ACS Omega. 2026 Jul 3;11(28):42631–42. doi: 10.1021/acsomega.6c03824 (PMC13392880; doi:10.1021/acsomega.6c03824)
Supplement: Supplementary file 1 [file ao6c03824_si_001.pdf]

Sequential Ion-Exchange Polishing of Oil Palm Trunk-Derived XOS-Containing Liquor: Effects of Holding Time and Water Washing on Sugar Recovery and Impurity Removal

Che Engku Noramalina Che Engku Chik, [a,b,‡]*, Yitong Niu,* [a,b,‡]*, Rozi Nuraika Ramli,* [a,b,c]*, Joo Shun Tan* [a,b]*, and Chee Keong Lee* [a,b*]*.*

[a] Bioprocess Technology Division, School of Industrial Technology, Universiti Sains Malaysia, 11800 Gelugor, Pulau Pinang, Malaysia

[b] Renewable Biomass Transformation Cluster, School of Industrial Technology, Universiti Sains Malaysia, 11800 Gelugor, Pulau Pinang, Malaysia

[c] Malaysia Institute of Pharmaceuticals and Nutraceuticals, National Institutes of Biotechnology Malaysia, Halaman Bukit Gambir, 11700 Gelugor, Penang, Malaysia

Supplement Figures

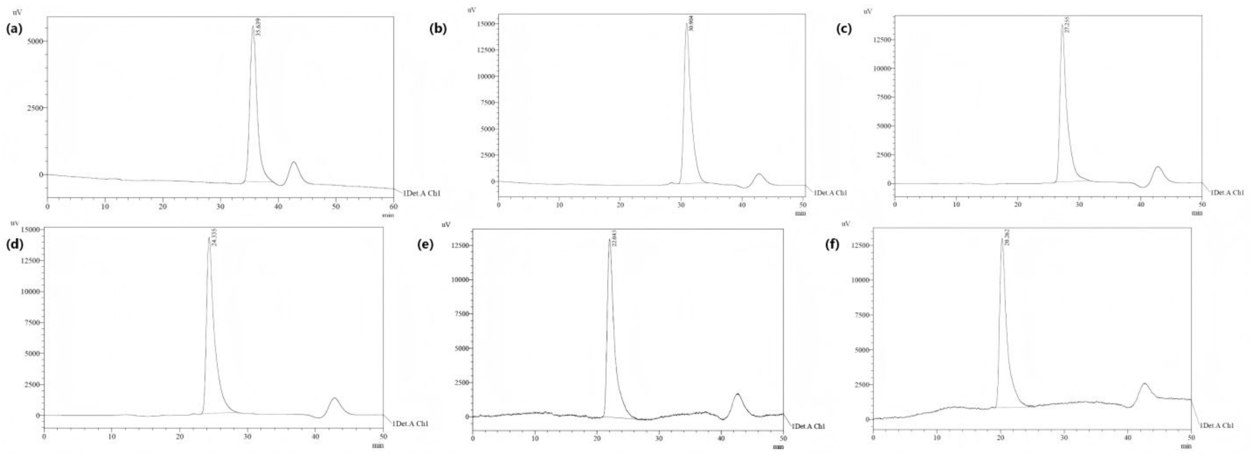

Figure S1. HPLC chromatograms of xylose and DP2–DP6 XOS standards: (a) Xylose, (b) xylobiose (DP2), (c) xylotriose (DP3), (d) xylotetraose (DP4), (e) xylopentaose (DP5), and (f) xylohexaose (DP6). The individual standards were analyzed under the same chromatographic conditions used for process samples.

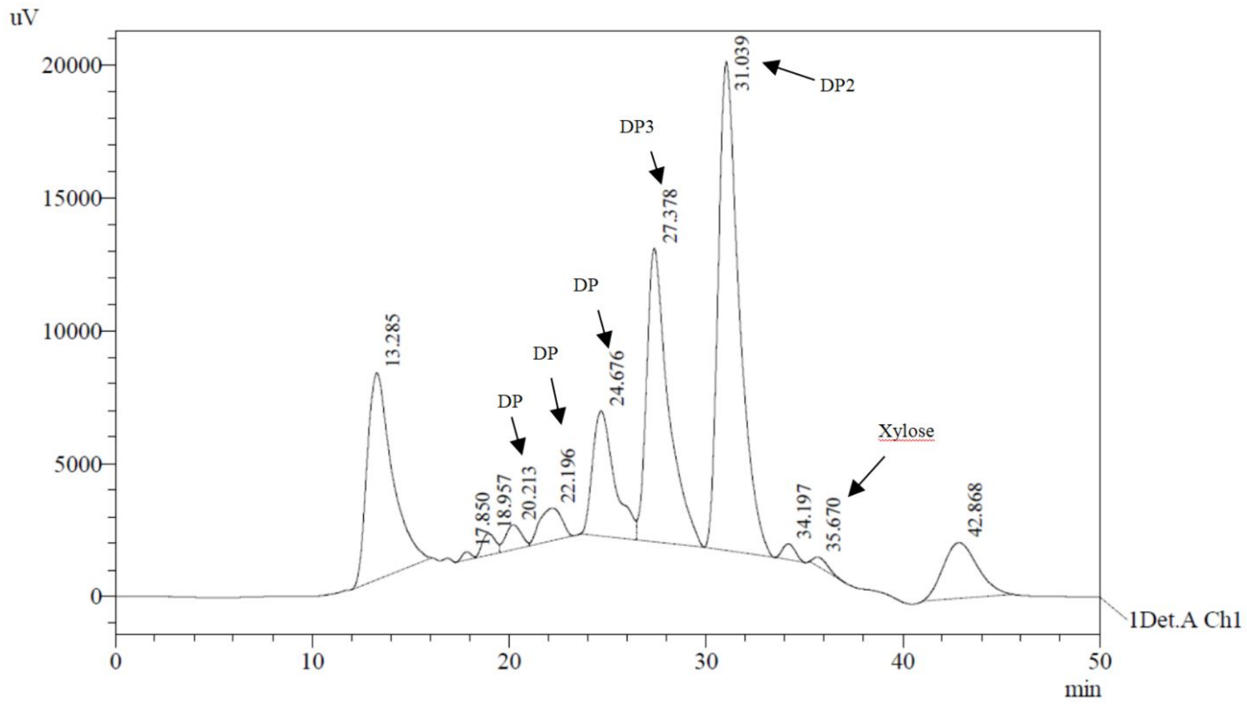

Figure S2. HPLC chromatogram of the commercial XOS reference.

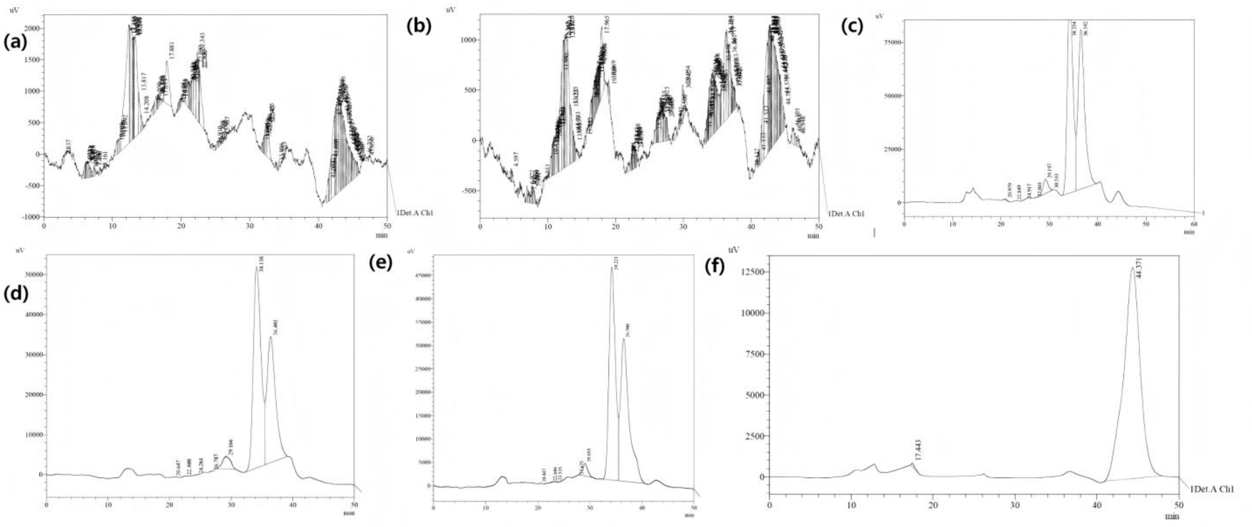

Figure S3 Representative HPLC chromatograms of OPT-derived process streams during XOS production and purification: (a) Autohydrolysate, (b) enzymatic lysate, (c) activated-carbon-purified fraction, (d) anion-exchange fraction, (e) cation-exchange fraction, and (f) waste-unbound fraction.

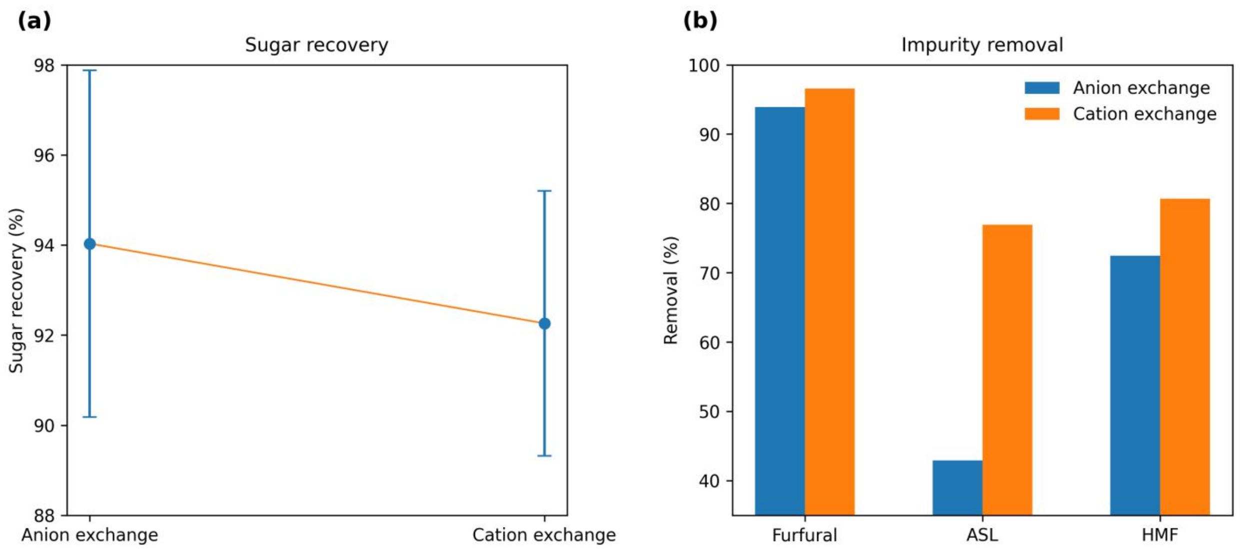

Figure S4. Effect of post-elution water washing under the optimized ion-exchange condition: (a) sugar recovery and (b) impurity removal.

Supplement Tables

Table S1. Retention times of xylose and DP2–DP6 XOS standards determined by HPLC.

No	Saccharides	Retention time (min)
1	Xylose (monosaccharides)	35.639
2	Xylobiose (DP2)	30.904
3	Xylotriose (DP3)	27.255
4	Xylotetraose (DP4)	24.335
5	Xylopentaose (DP5)	22.043
6	Xylohexose (DP6)	20.262

Note: Retention times were obtained from individual standards analyzed under the same HPLC conditions as the OPT-derived process samples.

Table S2. HPLC peak assignment and quantification of xylose and DP2–DP6 XOS in standards, commercial XOS, and OPT-derived process streams.

Sample	Saccharide	Retention time (min)	Peak area	Concentration (mg/mL)	Amount (g)
Standard	Xylose	35.639	1309544	1.000	—
Standard	DP2	30.904	1243557	1.000	—
Standard	DP3	27.255	1088126	1.000	—
Standard	DP4	24.335	1157353	1.000	—
Standard	DP5	22.043	1056807	1.000	—
Standard	DP6	20.262	982413	1.000	—
Commercial XOS	Xylose	35.670	19086	0.020	—
Commercial XOS	DP2	31.039	1366902	1.100	—
Commercial XOS	DP3	27.378	851362	0.780	—
Commercial XOS	DP4	24.676	368527	0.320	—
Commercial XOS	DP5	22.196	96125	0.100	—
Commercial XOS	DP6	20.213	51225	0.050	—
Autohydrolysate	Xylose	36.796	383	0.009	0.141
Autohydrolysate	DP2	31.765	1226	0.028	0.449

Sample	Saccharide	Retention time (min)	Peak area	Concentration (mg/mL)	Amount (g)
Autohydrolysate	DP4	24.910	1154	0.032	0.509
Autohydrolysate	DP5	22.092	15335	0.435	6.965
Autohydrolysate	DP6	20.971	7039	0.215	3.439
Lysate	Xylose	36.284	16264	0.373	5.961
Lysate	DP2	30.042	2864	0.069	1.105
Lysate	DP3	27.263	3368	0.093	1.486
Lysate	DP4	25.700	1871	0.048	0.776
Lysate	DP5	22.342	1378	0.039	0.626
Lysate	DP6	19.100	1663	0.034	0.542
XOS-rich, AC	Xylose	36.542	9195539	70.219	245.768
XOS-rich, AC	DP2	29.197	507927	4.084	14.296
XOS-rich, AC	DP3	27.041	1037	0.010	0.033
XOS-rich, AC	DP4	24.917	106	0.001	0.003
XOS-rich, AC	DP5	22.849	268	0.003	0.009
XOS-rich, AC	DP6	20.979	20302	0.207	0.723
XOS-rich, anion	Xylose	36.401	3929169	45.007	202.528
XOS-rich, anion	DP2	29.166	491521	5.929	26.680
XOS-rich, anion	DP3	26.787	3462	0.048	0.215
XOS-rich, anion	DP4	24.261	1246	0.016	0.073
XOS-rich, anion	DP5	22.400	919	0.013	0.059
XOS-rich, anion	DP6	20.647	927	0.014	0.064
XOS-rich, cation	Xylose	36.500	3329053	35.590	195.745
XOS-rich, cation	DP2	29.055	587585	6.615	36.383
XOS-rich, cation	DP3	27.675	909	0.012	0.064
XOS-rich, cation	DP4	23.535	1042	0.013	0.069
XOS-rich, cation	DP5	22.646	6185	0.082	0.451
XOS-rich, cation	DP6	20.447	171	0.002	0.013

Notes: Peak assignment was based on retention-time matching with individual xylose and DP2–DP6 standards analyzed under the same HPLC conditions. Amounts were calculated from the

measured concentrations and the corresponding process-stream volumes. AC, activated carbon; DP, degree of polymerization; XOS, xylooligosaccharides.
